# Short-Term Effects of a Structured Boxing Program on Technical Skill Acquisition in Novice Female Students

**DOI:** 10.3390/jfmk11010026

**Published:** 2026-01-08

**Authors:** Francesca Martusciello, Andrea Perazzetti, Arben Kaçurri, Sead Bushati, Aldo Muçalliu, Antonio Tessitore

**Affiliations:** 1 Department of Movement, Human and Health Sciences, University of Rome “Foro Italico”, 00135 Rome, Italy; francesca.martusciello@uniroma4.it; 2 National PhD Programme in Kinesiology and Sport Sciences, Department of Neurosciences, Biomedicine and Movement, University of Verona, 37129 Verona, Italy; 3 Degree Course in Sciences of Motor Activities, Sports and Psychomotor Education, Faculty of Law, Telematic University Giustino Fortunato, 82100 Benevento, Italy; a.perazzetti@unifortunato.eu; 4 Sports Research Institute, Sports University of Tirana, 1001 Tirana, Albania; akacurri@ust.edu.al (A.K.); sbushati@ust.edu.al (S.B.); amucalliu@ust.edu.al (A.M.)

**Keywords:** boxing education, motor learning, skill acquisition, female students, technical performance

## Abstract

**Background:** Despite the increasing interest in combat sports within higher education, studies on technical skill acquisition among novice female students remains limited. This study examined the effects of a short-term structured boxing program on the acquisition and retention of fundamental technical skills, focusing on stance (S), straight punches (SP), hooks (H), and uppercuts (U) among novice female university students. **Methods**: Technical performance was assessed under static and dynamic conditions at baseline (T1), after four weeks of course (T2), and at a two-month follow-up (T3) using customized scoring system. Handgrip strength (HG) and countermovement jump (CMJ) were measured as exploratory neuromuscular outcomes. **Results**: Results showed significant improvements in all technical skills at T2 compared with T1, in both static and dynamic executions (*p* < 0.001). Straight punches showed higher composite scores than hooks and uppercuts, while static performance was superior to dynamic execution (*p* < 0.001). Compared with T2, T3 showed a partial decline in performance for each skill in both executions (*p* < 0.001) (S_static_ = −18%; SP_static_ = −17%; H_static_ = −19%; U_static_ = −19%; S_dynamic_ = −22%; SP_dynamic_ = −18%; H_dynamic_ = −19%; U_dynamic_ = −21%), although T3 values generally remained above T1 baseline (S_static_ = +3%; SP_static_ = +19%; H_static_ = +22%; U_static_ = +29%; S_dynamic_ = −7%; SP_dynamic_ = +29%; H_dynamic_ = +29%; U_dynamic_ = +31%)**.** HG showed a significant time effect (*p* = 0.005), while CMJ did not change significantly. **Conclusions**: These findings indicate that a short-term structured boxing program can effectively improve the technical boxing skills in female beginners. This supports the inclusion of a boxing course in university sport science curricula to enhance technical, motor, and educational development.

## 1. Introduction

In general, the acquisition of motor and technical skills in combat sports follows recognizable developmental patterns. For instance, research on youth taekwondo practitioners has shown that coordination-related abilities, including reaction time, agility, and technical precision, improve significantly with age and technical experience [1]. Notably, female athletes achieved higher scores in certain technical skill tests, suggesting potential gender-specific learning advantages in specific movement patterns. These findings are particularly relevant for the design of training interventions targeting female participants, as they indicate that sex may influence both the rate and quality of technical skill development.

Boxing is a dynamic combat sport characterized by the continuous exchange of offensive and defensive actions [2]. Successful performance relies on an athlete’s ability to coordinate technical, physical, and tactical skills in real time. Achieving proficiency in boxing requires the integrated development of technical execution, physical conditioning, and motor coordination, enabling athletes to efficiently apply fundamental skills under competitive conditions [3]. Fundamental competencies in boxing include adopting a stable stance, maintaining balance during movement, and correctly executing the basic punches such as the jab, cross, hook, and uppercut [4]. Mastering these key techniques is crucial in the early stages of training, as they lay the groundwork for more complex technical and tactical skills [5,6].

Boxing engages practitioners in a learning process that extends beyond physical performance, fostering resilience and adaptability. Central to this is the development of self-efficacy, which has been shown to enhance well-being and social connectedness in community-based programs [7]. Taken together, the physical, psychological, and social benefits of boxing highlight its potential as more than just a sport, positioning it as an educational resource capable of supporting both personal development and structured learning processes. Consistent with this perspective, recent evidence indicates that integrating combat sports, such as boxing, into higher education can enhance student engagement, self-discipline, and academic achievement, while also strengthening social connectedness [8]. In fact, according to a review by Mathunjwa [8], combat sports offer a structured framework that integrates physical development with psychological and social competencies, thereby supporting the holistic growth of students. Moreover, the authors emphasized the importance of inclusive programming that addresses the needs of diverse student populations, including those from underrepresented backgrounds.

In recent years, combat sports have been incorporated into physical education programs to boost students’ motivation and improve learning quality, as well as into university curricula to enhance undergraduate students’ subjective well-being and self-esteem [9]. In this context, boxing serves as a valuable example of this approach, as it promotes not only physical fitness but also technical, psychomotor, and psychosocial development among students [10]. Several countries have introduced boxing as part of university physical education programs, with notable benefits. For instance, a recent study on first-year university students showed that boxing training significantly improved their overall physical fitness, including increases in strength, speed, endurance, and coordination compared with traditional physical education programs [11]. Similarly, a six-week “shadow boxing program” resulted in statistically significant reductions in body fat percentage among university students, highlighting the effectiveness of this practice as an accessible means of promoting health and preventing obesity [12]. Furthermore, studies focusing specifically on female students have confirmed the benefits of integrating boxing into higher education curricula. In particular, a one-year program comprising two 90-min sessions per week produced significantly greater improvements in students’ functional capacities and motor abilities compared to a control group following the traditional curriculum, thereby supporting the value of boxing as an innovative pedagogical tool in higher education [10]. These findings reinforce the practicality of incorporating boxing into existing physical education and sport science curricula as an effective program to enhance skill acquisition, physical development, and student engagement.

Nonetheless, notable gaps remain within the current body of literature, particularly regarding female student participants and short-term training interventions. Although previous research has documented physiological and performance differences between male and female boxers [13,14], limited attention has been given to the mechanisms underlying technical skill acquisition among female novices. Most existing studies have focused on long-term adaptations or elite-level performance [4,15], providing only a partial understanding of the early learning processes and the consolidation of fundamental techniques in beginners. Addressing this gap is essential for developing evidence-based guidelines for integrating boxing into university curricula. Indeed, from an educational perspective, there is limited evidence on the effects of a short-term, structured boxing program delivered within higher education, particularly targeting female university students with no prior boxing experience. In this context, a combined assessment of technical execution under static and dynamic conditions, along with a follow-up evaluation, would enable the examination of both initial skill acquisition and early retention. This approach would provide valuable insights into technical learning processes within time-limited educational interventions.

On this basis, it was hypothesized that participation in a short-term structured university boxing program would improve the fundamental boxing techniques in novice female sport science students.

To this end, the present study aims to examine the development of fundamental boxing techniques, including the boxing stance and the execution of basic punches (jab, cross, hook, and uppercut), in both static and dynamic conditions, among novice female sport science students participating in a university short-term structured boxing program.

## 2. Materials and Methods

### 2.1. Experimental Design

This investigation employed a longitudinal, repeated-measures design to assess the effects of a short-term technical boxing program on novice female students. The boxing program for novices used in our study was specifically designed based on the standard program of the University of Tirana’s course. It aimed to teach basic boxing technical skills and was revised according to the existing literature on boxing education and motor learning [10]. The intervention consisted of eight supervised sessions (two per week, each lasting 90 min) over a four-week period, focusing on fundamental boxing skills such as S, SP, H, and U. These skills were then assessed both in static (executed from a fixed stance) and dynamic (executed with displacement) conditions during three time points: (a) baseline, pre-training (T1); (b) after four weeks (T2); and (c) as follow-up, two months after the end of the program (T3). During the follow-up period, participants did not receive specific instructions and were asked to maintain their usual academic and daily activities. Engagement in other physical activities during this period was not systematically monitored.

### 2.2. Subjects

Out of an initial cohort of twenty-five female students from the Sports University of Tirana, nineteen of them (age: 20.0 ± 0.6 years, range: 19–21) engaged in a short-term structured boxing program. Six participants were excluded from the final analysis for not meeting the minimum adherence threshold of 85% attendance to the lessons.

Although all participants were undergraduate students enrolled in a Sport Science degree program and were physically active as part of their academic curriculum, none had prior boxing experience. However, information regarding their general sports background or habitual physical activity was not systematically collected and, therefore, could not be controlled for in the analysis. Exclusion criteria included any current or recent musculoskeletal injuries (upper or lower limbs) within the previous six months, cardiovascular or neurological disorders, or any medical condition contraindicating moderate-to-vigorous physical activity. The study was conducted in accordance with the Declaration of Helsinki and received approval from the Ethics Committee of the Sports University of Tirana (Prot. No. 1514/2), ensuring compliance with all applicable guidelines, regulations, and ethical standards for research involving human participants. All participants provided written informed consent prior to their enrollment. To guarantee anonymity, personal information was coded.

### 2.3. Procedures

The nineteen female students attended eight individual boxing sessions, each following the same structure, which included a 10-min warm-up phase (light jogging, joint mobility, and dynamic stretching), a 60-min technical drills, and a 10–15-min cool-down. During the first two weeks, technical practice was focused primarily on static execution, emphasizing the guard position and the correct mechanics of straight punches, hooks, and uppercuts. In the subsequent two weeks, dynamic execution was gradually introduced, incorporating displacement and coordination of footwork with punching actions. Each 60-min technical block included shadow boxing in front of a mirror for self-observation and movement control, and practice with paired drills. Among these, shoulder tag drills were used as playful exercises to develop reaction timing, defensive awareness, and spatial perception in a relaxed setting. Every session concluded with light stretching and breathing exercises to promote recovery and relaxation. To provide a clear and replicable description of the short-term structured boxing program, the content of the training sessions is summarized in Table 1.

The organization of the university boxing program in our study comprised three classes, each consisting of both female and male students. The didactic structure included theoretical and practical lessons. While the theoretical component was taught to both sexes, the practical lessons were conducted separately for each gender to ensure optimal supervision. Of the 25 female students considered, only 19 met the inclusion criteria; thus, the three classes were composed of 6, 6, and 7 female students, respectively. Each session content was then replicated three times and supervised by a certified boxing coach from the Albanian Boxing Federation, who has an academic background in sports sciences and previous experience teaching boxing fundamentals to beginners.

A customized scoring system to evaluate basic boxing technical skills, named “Boxing Novice Posture and Technique Evaluation” (BNPTE) [16], was used to assess the technical execution of the boxing stance and three punching techniques (i.e., straights, hooks, and uppercuts), performed both in static and dynamic conditions. Participants’ performances were evaluated using the four subscales of the BNPTE: (1) Stance (S); (2) Straight punch techniques (SP); (3) Hook punch techniques (H); (4) Uppercut punch techniques (U). All punches were performed with both lead and rear hands. Subscale 1 consisted of five items: shoulder, chin, body weight, feet and hands. Subscales 2–4 each consisted of four 4 items: on guard position, execution, trajectory, and body weight. All items across the four subscales were evaluated using a 4-point Likert scale, where 1 indicates poor execution, 2 fair, 3 good, and 4 very good. Ratings were summed to obtain a composite score for each technical skill. The evaluation was conducted separately for S, SP, H, and U. The sum of the ratings provided a composite score for each skill. One of the authors, who is qualified as a boxing coach by the Italian Boxing Federation, carried out the evaluation. All performances were evaluated twice by the same researcher: first in real time and then from video recordings using slow-motion analysis with Kinovea (version 0.9.5). The intra-observer reliability for the real-time and re-scoring analysis was high (ICC = 0.93).

In addition to the boxing skills assessments, two neuromuscular tests, such as handgrip strength (HG) and the countermovement jump (CMJ), were included as exploratory measures to provide complementary insights into overall physical performance. HG was assessed using a calibrated digital dynamometer (Lode, Groningen, The Netherlands), with two trials performed for the dominant hand at each time point (T1, T2 and T3). Participants were seated with their elbows flexed at 90°, forearms in a neutral position, and wrists in slight extension (0–30°) [17]. The best value obtained from the trials was retained for analysis. CMJ was assessed using the Leonardo Mechanograph^®^ jump platform (Novotec Medical GmbH, Pforzheim, Germany), which records ground reaction forces and computes jump parameters with high accuracy. Participants performed two CMJ trials with their hands on their hips to minimize arm contribution. Each jump was preceded by a standardized warm-up and separated by at least 60 s of passive recovery. The highest jump height recorded was retained for analysis.

### 2.4. Statistical Analysis

After setting the level of significance at 0.05, all statistical analyses were performed using the Jamovi software (version 2.6.44, x64; Jamovi, Sydney, Australia). The Q–Q plots were used for the initial graphical inspection of data distribution, and the normality was further assessed using the Shapiro–Wilk test, which indicated that most variables were normally distributed. Consequently, the combined graphical and numerical evaluations, along with the robustness of repeated-measures ANOVA in balanced within-subject designs, supported the use of parametric analyses in this study. Descriptive statistics included means and standard deviations (SD). A 2 (condition: static, dynamic) × 3 (time: T1: pre-training, T2: post-training, T3: follow-up) repeated measures ANOVA was applied separately to each technical skill (S, SP, H, and U). Post hoc pairwise comparisons were adjusted using the Bonferroni correction.

Regarding HG and CMJ, a repeated-measures ANOVA with three time points (T1: pre-training, T2: post-training, T3: follow-up) was conducted.

## 3. Results

Descriptive statistics of the collapsed composite scores for each technical skill (S, SP, H and U), calculated separately for the static and dynamic conditions across the three assessment time points (T1, T2, T3).

Across all four skills (S, SP, H, and U), scores displayed a consistent temporal pattern, with a significant increase from T1 to T2 followed by a reduction at T3 in both static and dynamic conditions.

For the S, performance increased from T1 to T2 and decreased at T3 in both conditions. In the static condition, scores increased from 14.8 ± 0.63 at T1 to 18.7 ± 2.00 at T2 and decreased to 15.3 ± 3.77 at T3, while in the dynamic condition, values increased from 14.4 ± 2.11 to 17.1 ± 1.79 and decreased to 13.4 ± 2.81. For the SP, a similar temporal pattern was observed. Static performance increased from 10.3 ± 2.33 at T1 to 14.7 ± 2.00 at T2 and declined to 12.3 ± 2.18 at T3, while dynamic performance increased from 8.58 ± 1.89 to 13.5 ± 2.61 and decreased to 11.1 ± 2.32. For the H, static scores increased from 9.21 ± 2.74 at T1 to 13.8 ± 2.32 at T2 and decreased to 11.2 ± 2.64 at T3, whereas dynamic scores increased from 7.89 ± 2.03 to 12.3 ± 2.58 and declined to 9.95 ± 2.50. For the U, static scores increased from 8.37 ± 2.59 at T1 to 13.4 ± 2.99 at T2 and decreased to 10.8 ± 2.88 at T3. In the dynamic condition, scores increased from 7.32 ± 2.29 to 12.1 ± 2.83 and declined to 9.58 ± 2.34.

Across all time points, SP consistently showed higher composite scores compared with H and U, in both static and dynamic conditions.

Despite this decrease, the values at T3 remained higher than baseline, with the exception of the stance in the dynamic condition (Sstatic = +3%; SPstatic = +19%; Hstatic = +22%; Ustatic = +29%; Sdynamic = −7%; SPdynamic = +29%; Hdynamic = +29%; Udynamic = +31%), where no difference was detected between T1 and T3 (*p* = 1.0).

**Figure 1:**
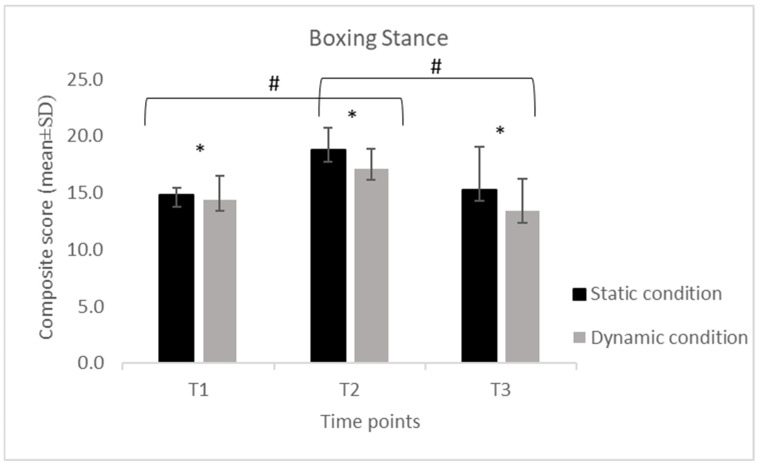
Mean (±SD) composite scores for the boxing stance across time points (T1: pre-training; T2: post-training; T3: follow-up) in static and dynamic conditions. Note. *: indicates a significant difference between static and dynamic conditions at all three time points (*p* < 0.001); #: indicates a significant difference between time points (*p* < 0.001).

**Figure 2:**
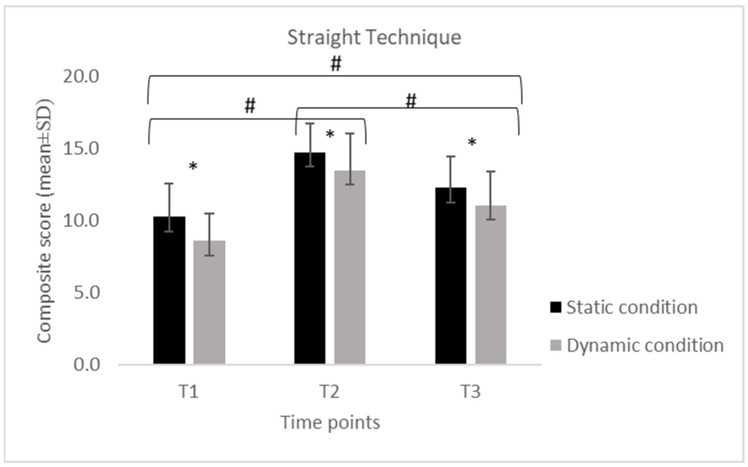
Mean (±SD) composite scores for the straight punch technique across time points (T1: pre-training; T2: post-training; T3: follow-up) in static and dynamic conditions Note. *: indicates a significant difference between static and dynamic conditions at all three time points (*p* < 0.001); #: indicates a significant difference between time points (*p* < 0.001).

**Figure 3:**
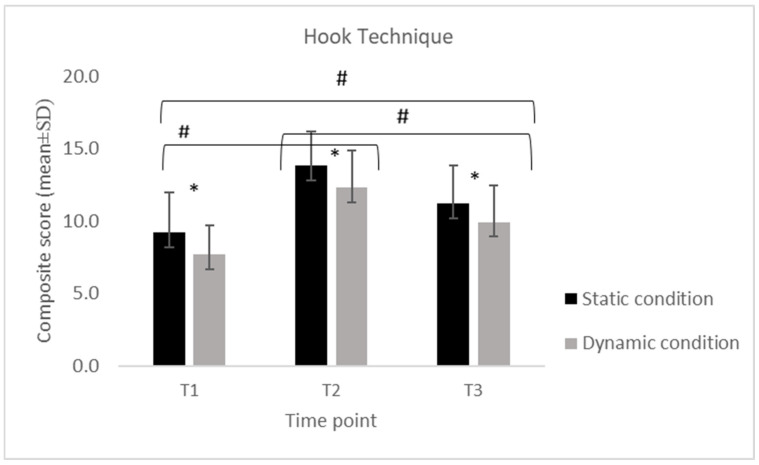
Mean (±SD) composite scores for the hook technique across time points (T1: pre-training; T2: post-training; T3: follow-up) in static and dynamic conditions. Note. *: indicates a significant difference between static and dynamic conditions at all three time points (*p* < 0.001); #: indicates a significant difference between time points (*p* < 0.001).

**Figure 4:**
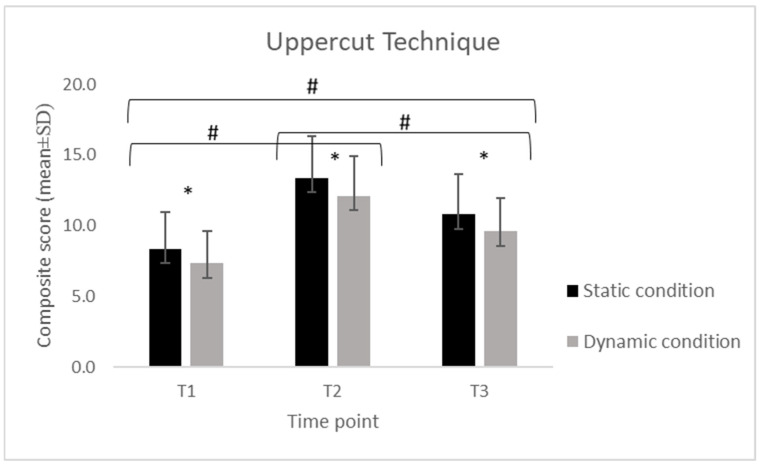
Mean (±SD) composite scores for the uppercut technique across time points (T1: pre-training; T2: post-training; T3: follow-up) in static and dynamic conditions. Note. *: indicates a significant difference between static and dynamic conditions at all three time points (*p* < 0.001); #: indicates a significant difference between time points (*p* < 0.001).

The 2 × 3 repeated-measures ANOVA revealed significant differences over time for all skills (S: F = 32.70, *p* < 0.001; SP: F = 53.58, *p* < 0.001; H: F = 57.19, *p* < 0.001; U: F = 75.79, *p* < 0.001). The Bonferroni post hoc comparisons confirmed that scores at T2 were significantly higher than those at both T1 and T3 (all *p* < 0.001), while no difference emerged between T1 and T3 for stance (Figure 1, Figure 2, Figure 3 and Figure 4). A significant difference for condition was also found across all skills (S: F = 19.52, *p* < 0.001; SP: F = 29.78, *p* < 0.001; H: F = 58.98, *p* < 0.001; U: F = 31.10, *p* < 0.001), with consistently higher scores in the static compared to the dynamic execution (Figure 1, Figure 2, Figure 3 and Figure 4). No significant interactions between time and condition were observed.

Descriptive statistics for HG and CMJ height across the three time points (T1, T2, and T3) are reported in Table 2.

For HG, repeated-measures ANOVA revealed a significant difference for time (F = 6.23, *p* = 0.005), with Bonferroni post hoc tests indicating a difference between T2 and T3 (*p* = 0.002), while no differences were observed between T1 and T2 or between T1 and T3.

Regarding CMJ, repeated-measures ANOVA did not show a significant main effect of time on jump height (F = 1.23, *p* = 0.305).

## 4. Discussion

To the best of the authors’ knowledge, this is the first study conducted in a higher education setting to evaluate the effects of a short-term structured boxing program on the acquisition of basic boxing techniques, such as stance, straight punches, hooks, and uppercuts, among female university students with no prior boxing experience. This study employs a combined framework that assesses both static and dynamic execution and includes a two-month follow-up to investigate early retention patterns in novices, which together highlight the novelty of this work.

The main findings were as follows: (a) all types of punches showed significant improvements immediately after the short-term structured boxing program (T2) compared with baseline (T1), highlighting the effectiveness of a targeted intervention in promoting technical mastery; (b) SP were easier to perform correctly than H and U, in both in static and dynamic conditions; (c) overall performance was higher under static conditions than under dynamic ones, suggesting greater technical complexity associated with movement execution; (d) at follow-up (T3), a partial decline in performance was observed across all punch types, indicating that skill consolidation requires ongoing practice; (e) handgrip strength increased significantly after the intervention, whereas countermovement jump (CMJ) height did not show significant changes.

Overall, the results indicate that the short-term structured boxing program achieved the planned objective of improving the technical execution of fundamental punches in novice female boxers, as evidenced by the significant gains observed immediately after the intervention. However, while the program was effective in promoting initial skill acquisition, ongoing practice appears necessary to fully consolidate these skills, particularly the more complex techniques such as hooks and uppercuts, and to ensure the transfer of improvements to dynamic conditions.

In the context of motor learning, acquiring a new skill involves progressing through a predictable sequence of levels and stages, as described by different theoretical models [18]. These include the three-stage model by Fitts and Posner [19], which focuses on the learner’s cognitive state during the learning continuum, and the expanded three-level model with its associated stages by Gallahue [20]. Although originally introduced to explain motor development in children, Gallahue’s model introduces an initial beginning or novice level, where the learner develops a conscious mental plan for the skill’s demands based on awareness, exploratory, and discovery stages [21]. The technical advancements observed in our study can be viewed as indicative of the initial cognitive stage of skill acquisition, in which students progressively develop an understanding of task requirements and begin to construct more organized and coherent motor representations. Previous research explaining how the acquisition and retention of different skills involve basic mechanisms of neuronal plasticity in the adult brain, has demonstrated that learning new skills is acquired throughout several stages, with an initial “rapid acquisition” that results in an intra-session improvement phase, succeeded by a consolidation period lasting several hours duration, and subsequently a “gradual acquisition” phase characterized by delayed, incremental gains in performance [22]. Therefore, it is worth noting that the supervised nature of our intervention, which provided immediate corrective feedback and technical modeling, may have contributed to these improvements. 

The effectiveness of the short-term structured boxing program in our study is evidenced by the significant improvement in punches techniques (jab, cross, hook, and uppercut) among female novice students immediately after the training period (T2). This finding supports the notion that targeted, supervised interventions are effective in promoting motor learning in beginner boxers. This aligns with Bingül [6], who reported that focused practice of specific punches facilitates rapid development of accuracy and coordination. In our study an additional factor contributing to effectiveness was that the boxing lessons were taught by a former elite boxer who is certified as boxing coach by the Albanian Boxing Federation. The instructor also has an academic background as a professor at the Sport University of Tirana, with previous experience teaching boxing fundamentals to beginners. This aligns with Lindsay and Lenetsky’s [23] statement that experiential knowledge of (elite) coaches regarding whole-body movements, footwork, hip and shoulder rotation, and hand and arm positioning is effective in enhancing punching techniques in boxing.

In this study, SP (jabs and crosses) were found to be easier to execute correctly than H and U, both in static and dynamic conditions. This can be explained by their linear trajectory of these punches, which requires less rotation of the trunk and lower limbs, allowing for a more stable distribution of body weight during execution [3]. In contrast, hooks and uppercuts proved to be more difficult to execute, as they involve more circular movements and greater engagement of the trunk and hips, increasing technical complexity and the likelihood of execution errors [6,24]. These findings are consistent with previous studies, which have shown that punch biomechanics influence the ease of skill acquisition among beginners. The greater difficulty associated with hooks and uppercuts underscores the importance of repeated, supervised practice in consolidating technical competence, especially under dynamic conditions. These findings align with research on the neuromotor characteristics of young female boxers, which reported variable degrees of bilateral coordination, with some athletes exhibiting marked performance disparities between their right and left hands and only minimal overall correlations between their movements [25]. This evidence highlights the presence of individual asymmetries and variability in coordination, which could be speculated may have contributed to the difficulties encountered by our female student novices in executing complex techniques and performing under dynamic conditions. From this viewpoint, targeted training focused on improving bilateral coordination and trunk control may facilitate the acquisition of more complex punching techniques, especially those requiring extensive rotational movements and weight transfer.

Overall performance was higher under static conditions than under dynamic ones, suggesting that movement execution involves greater technical complexity when dynamic. The results may indicate that, in static conditions, athletes are better able to focus on posture, movement sequencing, and weight transfer, thereby reducing the number of motor and cognitive variables that need to be managed simultaneously. In contrast, performing movements dynamically may require a more complex integration of body displacement and motor coordination, potentially increasing both cognitive and motor loads. From this perspective, the transition from static to dynamic execution may represent a crucial phase in the technical learning process. This interpretation is supported by literature on motor control and dual-task performance. Recent studies have shown that performing complex motor tasks under dynamic conditions imposes a greater cognitive load than static execution, as it requires the simultaneous integration of multiple sensory inputs and the continuous adjustment of movement patterns [26]. These findings suggest that the ability to manage increased cognitive and motor demands during dynamic execution represents a critical stage in the technical learning process, marking the transition from controlled and conscious performance to more automatic and adaptable skill execution [18].

In line with this interpretation, the boxing program’s structure was specifically designed to progress from static to dynamic execution, as detailed in Table 1. The first two weeks focused on static practice, with the expected effects of improving postural alignment and stabilizing punch mechanics through repeated drills and individual feedback. Weeks 3–4 progressively introduced displacement to promote the integration of punches with footwork. Accordingly, the improvements observed from T1 to T2 across stance and punching techniques in both static and dynamic conditions appear consistent with the technical progression planned within the structured program.

Conversely, at the follow-up assessment (T3), a partial decline in performance was observed across all punch types, suggesting that after the initial learning phase, acquired skills tend to diminish in the absence of consistent reinforcement. The improvements recorded immediately after the training period can be interpreted in consideration of the power law of practice, which posits that learning occurs rapidly during the early stages of acquisition before gradually plateauing as performance stabilizes [27]. However, the subsequent phase, characterized by the lack of systematic practice, may lead to a progressive decay of skills, reflecting the limitations of short-term motor consolidation. In this regard, research on motor memory consolidation has shown that abilities acquired over brief practice periods are particularly susceptible to decay if not reinforced through additional practice sessions [28]. Despite the decline observed in our study at follow-up (T3), these values remained higher than at baseline (T1), except for the stance in the dynamic condition, where no difference was detected between T1 and T3. This finding aligns with literature that underscores how the quantity of practice alone does not influence the acquisition and retention of the task; rather, it is the distribution of practice over several days that is a key factor affecting learning and retention [29].

Finally, in boxing, isometric handgrip strength represents an important indicator of upper-limb strength and, more broadly, of the athlete’s overall physical condition. As reported by Chaabène [13], handgrip strength is considered a key component of performance in amateur boxing, as it contributes to wrist stability and punch control upon impact. In our study, HG showed variations over time, with a non-significant increase immediately after the end of the boxing course (T2), followed by a decline at the follow-up (T3). The absence of significant differences between T1 and T2 suggests that the observed increase may reflect a general trend rather than a consolidated effect of the intervention. The subsequent significant decrease at T3 indicates that this adaptation was transient and not maintained over time in the absence of continued practice. It can be hypothesized that the temporary improvement registered in our study in HG was primarily due to early neuromuscular adaptations rather than structural changes. During the initial stages of motor learning, increases in strength are often associated with enhanced intra- and intermuscular coordination, as well as more efficient recruitment of motor units [30]. Specifically, in the context of boxing, the repetitive execution of technical punches may indirectly stimulate the forearm musculature, leading to temporary improvements in contractile capacity. However, maintaining upper-limb strength likely requires more specific and prolonged training stimuli to produce stable structural adaptations, such as muscle hypertrophy and modifications in contractile properties. The decrease observed at T3 suggests that, in the absence of continuous practice, acute neural and tendinous adaptations tend to regress, highlighting the need for complementary strength training programs to accompany technical work and achieve lasting improvements.

In contrast, CMJ height did not show significant variations across the three testing sessions, suggesting that the eight lessons of the boxing course produced specific effects on the upper limbs rather than on lower-limb explosive power. This result appears consistent with the contents of the training program followed by our student novices, which focused primarily on technical punching drills that engage the upper limbs and trunk. Although boxing undeniably relies on lower-limb contribution for force generation and postural stability, the training program implemented in this study may not have provided sufficient stimulus to induce measurable adaptations in lower-limb explosive performance.

### Limitations and Future Directions

This study acknowledges some limitations. First, the relatively small and homogeneous sample, composed exclusively of novice female university sport science students from a single institution, limits the generalizability of the findings to broader populations or individuals with different levels of sporting experience. Second, the absence of a control group prevents a clear distinction between the specific effects of the structured boxing program and potential improvements resulting from general motor practice or increased familiarity with the testing procedures. Third, the four-week duration of the university course may have influenced the full consolidation and long-term retention of the acquired boxing technical skills.

Future studies should address these limitations by including larger and more diverse samples, incorporating control or comparison groups, and extending both the duration of training programs and follow-up assessments to better evaluate the long-term consolidation of technical abilities.

## 5. Conclusions

This longitudinal study demonstrates that a short-term, structured boxing program may have the potential to improve the technical execution of fundamental boxing skills, such as S, SP, H, and U, in novice female sport science students participating in a university beginner boxing course. Performance gains were evident immediately after the end of the training program (T2) in both static and dynamic conditions, with higher scores recorded under static execution. However, despite the partial decline observed at follow-up (T3) indicates that continuous practice is required to consolidate and maintain technical proficiency, these values remained higher than at baseline (T1).

The transient improvement in handgrip strength and the absence of significant changes in CMJ height suggest a specific neuromuscular adaptation of the upper limbs rather than an enhancement in lower-limb explosive power. Future training programs may benefit from incorporating complementary exercises designed to develop lower-limb strength and power, acknowledging their contribution to the kinetic chain of punching.

Overall, the findings of this study offer valuable insights for designing a short-term structured boxing program for beginners, particularly in educational or university settings. Even within limited timeframes, a well-structured and supervised program focused on fundamental techniques can effectively facilitate the acquisition and early consolidation of basic technical skills. Coaches and educators should maintain a balance between technical precision, progressive difficulty, and practice variability to optimize learning outcomes among novice practitioners.

## Figures and Tables

**Table 1:** Weekly structure and content of the university’s short-term structured boxing program.

Training Phase	Type of Drills	Teaching Method	Duration (min)	Main Technical Focus	Expected Effects
1st week					
Warm-up	Light jogging, mobility exercise.	Guided group activity	10	Activation and preparation	
Technical drills (static)	Boxing stance practice (feet alignment, weight distribution, hand and chin position) in a static condition	Demonstration and individual feedback	20	Postural stability and correct guard position	Improvement of postural alignment and continuous maintenance of the guard
Technical drills (static)	Straight punches (jab and cross) in a static condition	Repetitive drills	20	Linear trajectory, arm extension, opposite hand in guard	Acquisition of correct straight punch mechanics and control of return to guard
Technical drills (static)	Hooks and uppercuts (lead and rear) in a static condition	Guided technical drills	20	Trunk rotation, punch trajectory, body weight control	Development of basic coordination between trunk rotation and upper-limb actions
Play-based drill	Mirror drill (partner imitation exercise)	Exploratory practice	5	Paired coordination drill	
Cool-down	Light stretching and breathing exercises	Guided activity	10/15	Physical and mental recovery	
2nd week					
Warm-up	Light jogging, mobility exercise.	Guided group activity	10	Activation and preparation	
Technical drills (static)	Review and consolidation of boxing stance in a static condition.	Individual feedback	25	Stability and continuous chin protection	Reinforcement of postural stability and guard consistency
Technical drills (static)	Review and consolidation of straight punches, hooks and uppercuts (lead and rear) in a static condition.	Repetitive drills	25	Trajectory control and postural maintenance	Reduction in execution errors and increased movement consistency
Play-based drill	Mirror drill (partner imitation exercise)	Exploratory practice	15	Play-based coordination drill	
Cool-down	Light stretching and breathing exercises	Guided activity	10/15	Physical and mental recovery	
3rd week					
Warm-up	Light jogging, mobility exercise.	Guided group activity	10	Activation and preparation	
Technical drills (dynamic)	Straight punches (jab and cross) in a dynamic condition with forward and backward displacement.	Guided drills	25	Coordination between footwork and punches	Improved integration of punching actions with linear displacement
Technical drills (dynamic)	Hooks and uppercuts (lead and rear) in a dynamic condition with forward and backward displacement.	Guided drills	25	Coordination between footwork and punches	Enhanced trunk rotation control during movement
Paired drill	Shadow boxing with displacement	Exploratory practice	10	Motor organization in dynamic conditions	
Play-based drill	Shoulder tag	Exploratory practice	5	Play-based coordination drill	
Cool-down	Light stretching and breathing exercises	Guided activity	10/15	Physical recovery	
4th week					
Warm-up	Light jogging, mobility exercise.	Guided group activity	10	Activation and preparation	
Technical drills (dynamic)	Review and consolidation of boxing stance, straight punches, hooks and uppercuts (lead and rear) in a dynamic condition.	Variable practice	25	Trajectory control, postural maintenance and coordination between footwork and punches	Stabilization of technical execution under dynamic conditions
Technical drills (dynamic)	A combination of straight punches, hooks, and uppercuts (lead and rear) with displacement	Guided drills	25	Coordination and technical integration	Integration of multiple techniques and improved intersegmental coordination
Paired drill	Shadow boxing with displacement	Exploratory practice	10	Motor organization in dynamic conditions	
Play-based drill	Shoulder tag	Exploratory practice	5	Play-based coordination drill	
Cool-down	Light stretching and breathing exercises	Guided activity	10/15	Physical and mental recovery	

**Table 2:** Mean (±SD) values for handgrip strength (HG) and countermovement jump (CMJ) across time points (T1: pre-training; T2: post-training; T3: follow-up).

	T1 (Pre) (Mean ± SD)	T2 (Post) (Mean ± SD)	T3 (Follow-Up) (Mean ± SD)
HG	33.0 ± 4.65	35.3 ± 4.99 ^a^	32.5 ± 4.55
CMJ	38.1 ± 5.3	38.6 ± 4.4	39.6 ± 4.2

Note. ^a^: indicates a statistically significant difference compared with T3 (*p* = 0.002).

## Data Availability

Data are contained within the article.

## Abbreviations

The following abbreviations are used in this manuscript:

S	Stance
SP	Straight punches
H	Hook
U	Uppercut
HG	Hand Grip
CMJ	Countermovement Jump
BNPTE	Boxing Novice Posture and Technique Evaluation

## References

[1] Boutios S., Fiorilli G., Buonsenso A., Daniilidis P., Centorbi M., Intrieri M., Di Cagno A. (2021). The impact of age, gender and technical experience on three motor coordination skills in children practicing taekwondo. Int. J. Environ. Res. Public Health.

[2] Martusciello F., Perazzetti A., Kaçurri A., Consolati M., Tessitore A. (2025). Time–Motion Analysis of the 2023 Women’s World Boxing Championships Finals. Sports.

[3] Piorkowski B.A., Lees A., Barton G.J. (2011). Single maximal versus combination punch kinematics. Sports Biomech..

[4] Martusciello F., Perazzetti A., Kaçurri A., Consolati M., Tessitore A. (2025). Notational Analysis of the Final Matches of the 2023 IBA Women’s World Boxing Championships. J. Funct. Morphol. Kinesiol..

[5] Rakha A.H. (2024). Reflections on augmented reality codes for teaching fundamental defensive techniques to boxing beginners. PLoS ONE.

[6] Bingul B.M., Bulgun C., Tore O., Bal E., Aydin M. (2018). The effects of biomechanical factors to teach different hook punch techniques in boxing and education strategies. J. Educ. Train. Stud..

[7] Ryan A., John M., Hanna P. (2025). A community perspective on boxing, well-being and young people. J. Community Appl. Soc. Psychol..

[8] Mathunjwa M.L., Shaw I., Avramov D., Shaw B. (2025). Enhancing engagement and academic success through combat sports in higher education. Int. J. High. Educ..

[9] Ying L., Yang Q. (2025). The impact of combat sports on undergraduate students’ subjective well-being: Chain mediation effects of emotional intelligence and self-esteem. Front. Psychol..

[10] Kudryavtsev M., Kovalev V., Osipov A., Galimov G., Isaev R., Petukhova L., Karpenko E. (2023). Improving the physical health of female students using boxing specialization in physical education. J. Phys. Educ. Sport.

[11] Chuzhinov A.O., Timofeeva O.V., Lubyshev E.A., Fadina O.O. (2025). The impact of boxing training on the physical condition of first-year students. Theory Pract. Phys. Cult..

[12] Nugraha A.B.K., Roepajadi J., Kaharina A., Ilmi M.A., Ningsih Y.F. Effect of shadow boxing exercises on body fat percentage. Proceedings of the International Joint Conference on UNESA.

[13] Chaabène H., Tabben M., Mkaouer B., Franchini E., Negra Y., Hammami M., Hachana Y. (2015). Amateur boxing: Physical and physiological attributes. Sports Med..

[14] Davis P., Benson P.R., Waldock R., Connorton A.J. (2016). Performance analysis of elite amateur female boxers and comparison with their male counterparts. Int. J. Sports Physiol. Perform..

[15] Davis P., Benson P.R., Pitty J.D., Connorton A.J., Waldock R. (2015). The activity profile of elite male amateur boxing. Int. J. Sports Physiol. Perform..

[16] Martusciello F., Kaçurri A., Bushati S., Perazzetti A., Tessitore A. Development and Validation of the Boxing Novice Posture and Technique Evaluation scoring system: A new tool for basic boxing technical skills assessment. Proceedings of the XVII Forum of the European Network of Sport Education (ENSE).

[17] Adam C., Klissouras V., Ravazzolo M., Renson R., Tuxworth W. (1987). The Eurofit Test of European Physical Fitness Tests.

[18] Salehi S.K., Tahmasebi F., Talebrokni F.S. (2021). A different look at featured motor learning models: Comparison exam of Gallahue’s, Fitts and Posner’s and Ann Gentile’s motor learning models. Mov. Sport Sci.-Sci. Motricité.

[19] Fitts P.M., Posner M.I. (1967). Human Performance.

[20] Gallahue D.L. (1982). Assessing motor development in young children. Stud. Educ. Eval..

[21] Gallahue D.L., Ozmun J.C., Goodway J.D. (2012). Understanding Motor Development: Infants, Children, Adolescents, Adults.

[22] Karni A., Meyer G., Rey-Hipolito C., Jezzard P., Adams M.M., Turner R., Ungerleider L.G. (1998). The acquisition of skilled motor performance: Fast and slow experience-driven changes in primary motor cortex. Proc. Natl. Acad. Sci. USA.

[23] Lindsay R., Lenetsky S. (2020). The Contribution of Expert Coachesâ Experiential Knowledge in Understanding Punching Performance in Boxers. J. Emerg. Sports Stud..

[24] Kapo S., Kajmović H., Cutuk H., Beriŝa S. (2008). The level of use of technical and tactical elements in boxing based on the analysis of the 15th B&H individual boxing championship. Homo Sport..

[25] Tyshchenko V., Omelianenko H., Markova S., Vorontsov A., Pavelko O., Doroshenko E., Drobot K. (2023). Neurological typology and its role in enhancing technical and tactical skills in adolescent female boxers. Health Sport Rehabil..

[26] Saraiva M., Vilas-Boas J.P., Fernandes O.J., Castro M.A. (2023). Effects of Motor Task Difficulty on Postural Control Complexity during Dual Tasks in Young Adults: A Nonlinear Approach. Sensors.

[27] Newell A., Rosenbloom P.S. (2013). Mechanisms of skill acquisition and the law of practice. Cognitive Skills and Their Acquisition.

[28] Heydaripour S., Abdoli B., Shamsipour Dehkordi P. (2025). Practice distribution and self-talk effects on motor memory encoding and consolidation in unskilled adolescents. J. Mot. Behav..

[29] Savion-Lemieux T., Penhune V.B. (2005). The effects of practice and delay on motor skill learning and retention. Exp. Brain Res..

[30] Folland J.P., Williams A.G. (2007). Morphological and neurological contributions to increased strength. Sports Med..

